# Supplementary material for: High sensitivity analysis of nanogram quantities of glycosaminoglycans using ToF-SIMS
Source: Commun Chem. 2021 May 14;4:67. doi: 10.1038/s42004-021-00506-1 (PMC9814553; doi:10.1038/s42004-021-00506-1)
Supplement: Supplementary file 1 — Supplementary Information [file 42004_2021_506_MOESM1_ESM.pdf]

High sensitivity analysis of nanogram quantities of glycosaminoglycans using ToF-SIMS – Supplementary information

Andrew L. Hook[1]*, John Hogwood[2], Elaine Gray[2,3], Barbara Mulloy[3], Catherine L. R. Merry[4]

[1] Advanced Materials and Healthcare Technology, University of Nottingham, Nottingham NG7 2RD, UK
[2] National Institute for Biological Standards and Control, Potters Bar EN6 3QG, UK
[3] Institute for Pharmaceutical Science, King's College London, Franklin-Wilkins Building, Stamford Street, London SE1 9NH, UK
[4] Stem Cell Glycobiology Group, Biodiscovery Institute, Faculty of Medicine and Health Sciences, University of Nottingham, Nottingham NG7 2RD, UK

*Corresponding author: andrew.hook@nottingham.ac.uk

Supplementary Note 1. Microarray optimisation

Immobilisaton of GAGs to surfaces can be achieved by covalent and non-covalent methods. Numerous approaches have been explored to immobilise GAGs on surfaces including carbodiimide chemistry, divinyl sulfone activation, reductive amination, sulfhydryl-maleimide reactions, Diers-Alder reaction, azide, and diazirine chemistry[1-6]. To achieve non-covalent immobilisation, cationic surfaces are typically produced to enable ionic interactions[7-8]. Non-covalent interactions have the advantage of forming quickly (near instantaneous), effectively immobilising GAGs[7] in a biologically relevant manner[8]. Carbohydrate microarrays have emerged as useful tools for studying biomolecular interactions with glycans[6, 9-14], including the use of non-covalent interactions with Poly-L-lysine as an approach to rapidly and easily adhere GAGs to a surface[13].

To select an optimal substrate for forming a GAG microarray five different candidate surfaces were assessed, glass, poly-L-lysine coated glass slides, aminoalkyl silanised glass slides, tissue culture (TC) polystyrene and allylamine plasma polymer coated slides. Here we made use of non-covalent interactions due to the ease of formation and as the ToF-SIMS characterisation was not demanding on the GAG-substrate interaction.

1 µl of 2 mg/ml solutions of either HS or HA were manually pipetted onto each substrate and allowed to air dry. These two GAGs were selected in order to assess sulphated and non-sulphated biomolecules. The dried spots were then incubated overnight in 10 mls of water, before drying and analysis by ToF-SIMS. The intensity of both positively and negatively charged characteristic ions for the GAGs was compared on as-received materials, after deposition of GAGs and after washing (Supplementary Figure 1-2). For all substrates and for both GAGs the characteristic ions were low for as-received materials, and increased after the addition of either HS or HA. On almost all samples the characteristic ions reduced to similar intensities to the as-received materials after washing, suggesting that the washing procedure has completely removed the GAG. The exception to this was the cationic samples poly-L-lysine and allylamine plasma polymer, both of which maintained a higher intensity of characteristic ions for GAGs above baseline levels after washing. The PLL coated glass slide maintained a higher intensity of characteristic peaks for GAGs after washing compared with the allylamine plasma polymer coating and was thus selected for formation of subsequent arrays.

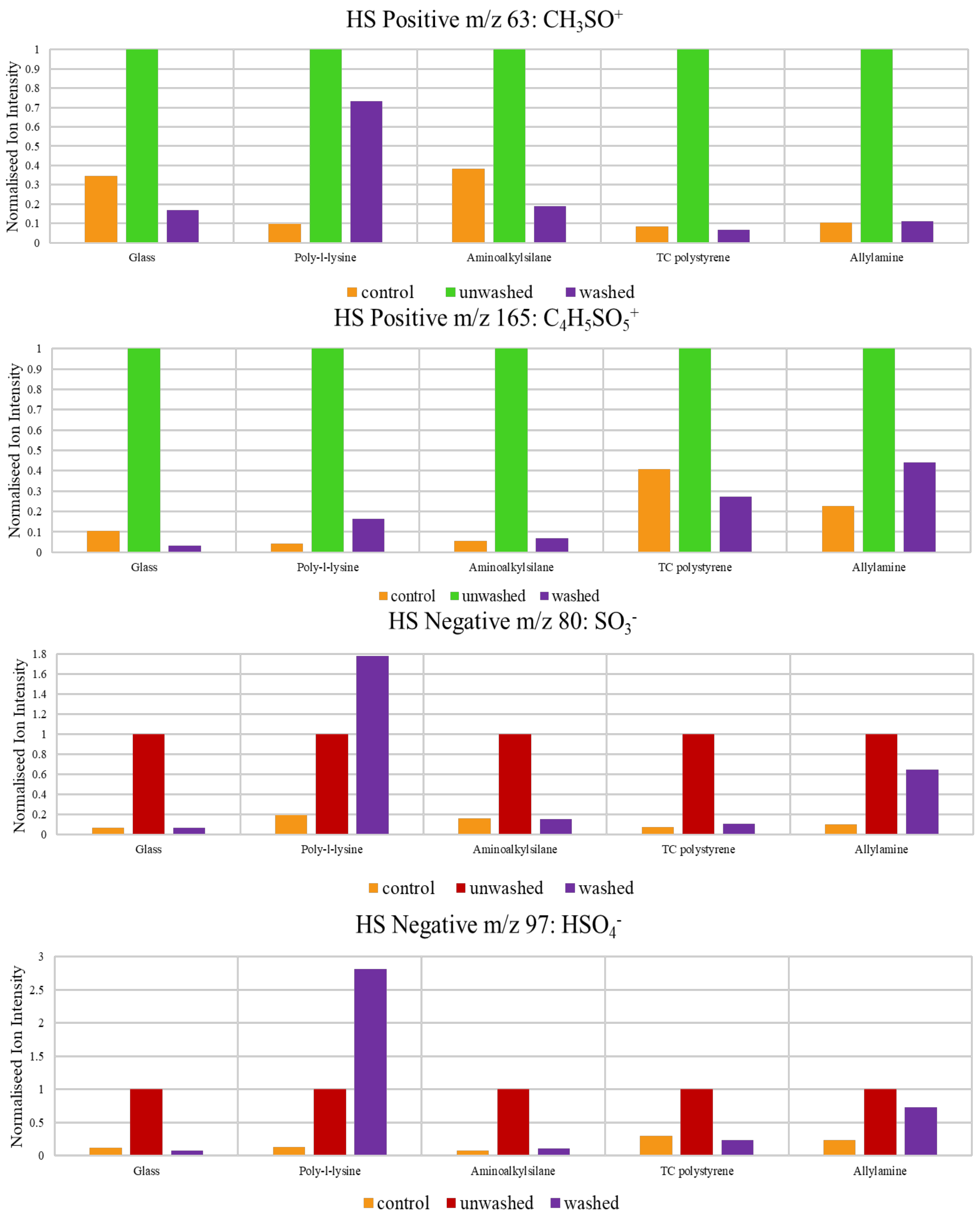

Supplementary Figure 1. Normalised ion intensities at specific mass/charge positions for glass, poly-l-lysine, aminosilane, TCPS, and allylamine plasma polymer before, and after washing and the addition of heparan sulphate (HS). The proposed mass assignment and m/z for the characteristic ions are shown.

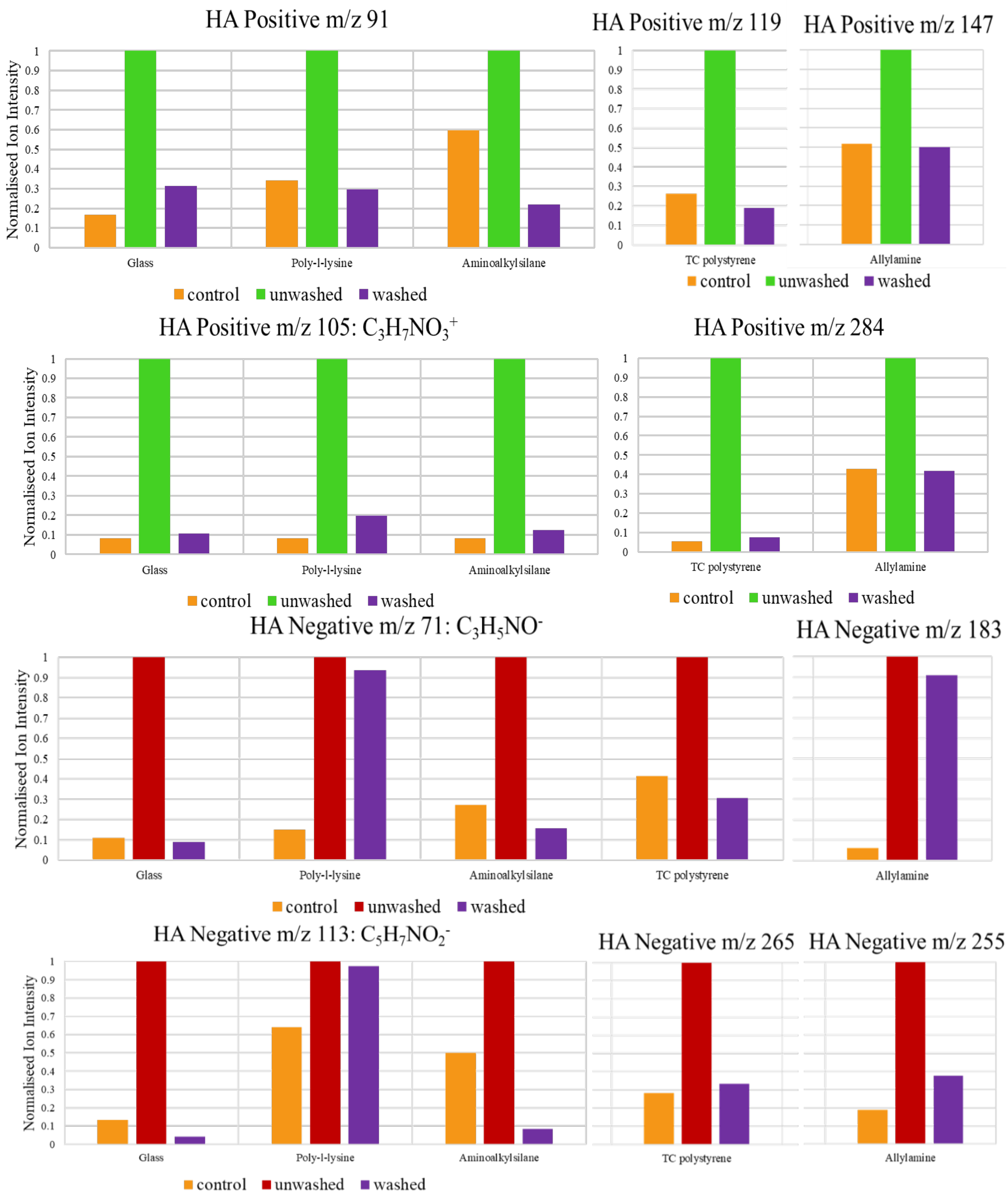

Supplementary Figure 2. Normalised ion intensities at specific mass/charge positions for glass, poly-l-lysine, aminosilane, TCPS, and allylamine plasma polymer before, and after washing and the addition of hyaluronic acid (HA). The proposed mass assignment and m/z for the characteristic ions are shown.

Supplementary Note 2. Demonstration of in-spot mixing

Use of ink-jet printing allows for the rapid creation of mixtures of samples using in-spot mixing[15]. To explore this with GAGs, HA, HS, CS and DS were printed at various combinations. To facilitate mixing, a 15 nL water droplet was initially printed and other GAGs were then sequentially deposited. Printing was done at 65 % humidity to reduce evaporation, and a sacrificial border of water droplets was printed around the array to create a local high humidity environment. Prior to printing the evaporation rate of droplets was determined. The calculated volume of water lost in each print

cycle was replaced in each print run by the use of two nozzles, one that delivered a GAG solution and the other that delivered water.

The resulting array is shown in Supplementary Figure 3. All spots were visible after drying. The array was analysed by SIMS and regions of interest were used to extract spectra for each spot. The resulting spectra were analysed by PCA to reduce the dimensionality of the dataset. A sparse feature dataset was created using recursive feature elimination using the total separation of the pure GAGs as a weighting (selection of feature to be eliminated was determined by the feature that would cause the largest separation of points in PC1 and PC2). The dataset was successfully reduced to 4 variables without a significant reduction in sample separation. The final scores plot for PC1 and PC2 are shown in Supplementary Figure 4. For each of the single GAGs, 7 repeats were used for the PCA analysis, whist 3 samples were used as a test set. The PCA showed that the 4 single GAGs were readily chemically discernible, with the clusters associated with replicates of each GAGs being clearly distinct. In all cases the test sets fell within the 95 % confidence ellipses determined for the training samples, suggesting that the variance captured by PC1 and 2 was real and the data had not been over-fitted. GAG mixed samples, when plotted onto the scores spot, fell outside of ellipses determined for the single GAG samples, however, samples were not observed to quantitatively fall between the ellipses for the single samples. This is expected due to matrix effects compromising the quantitative nature of SIMS data[16].

The 4 ions used for the PCA, possible assignments and loadings for PC1 and PC2 are shown in Supplementary Table 1. Apart from the ion SNO-, the assignment of the ions is ambiguous due to the mass resolution of ToF-SIMS. However, a number of possible assignments including sulphur, suggesting that the variance captured by the PCA is associated with the sulphation pattern of the GAGs.

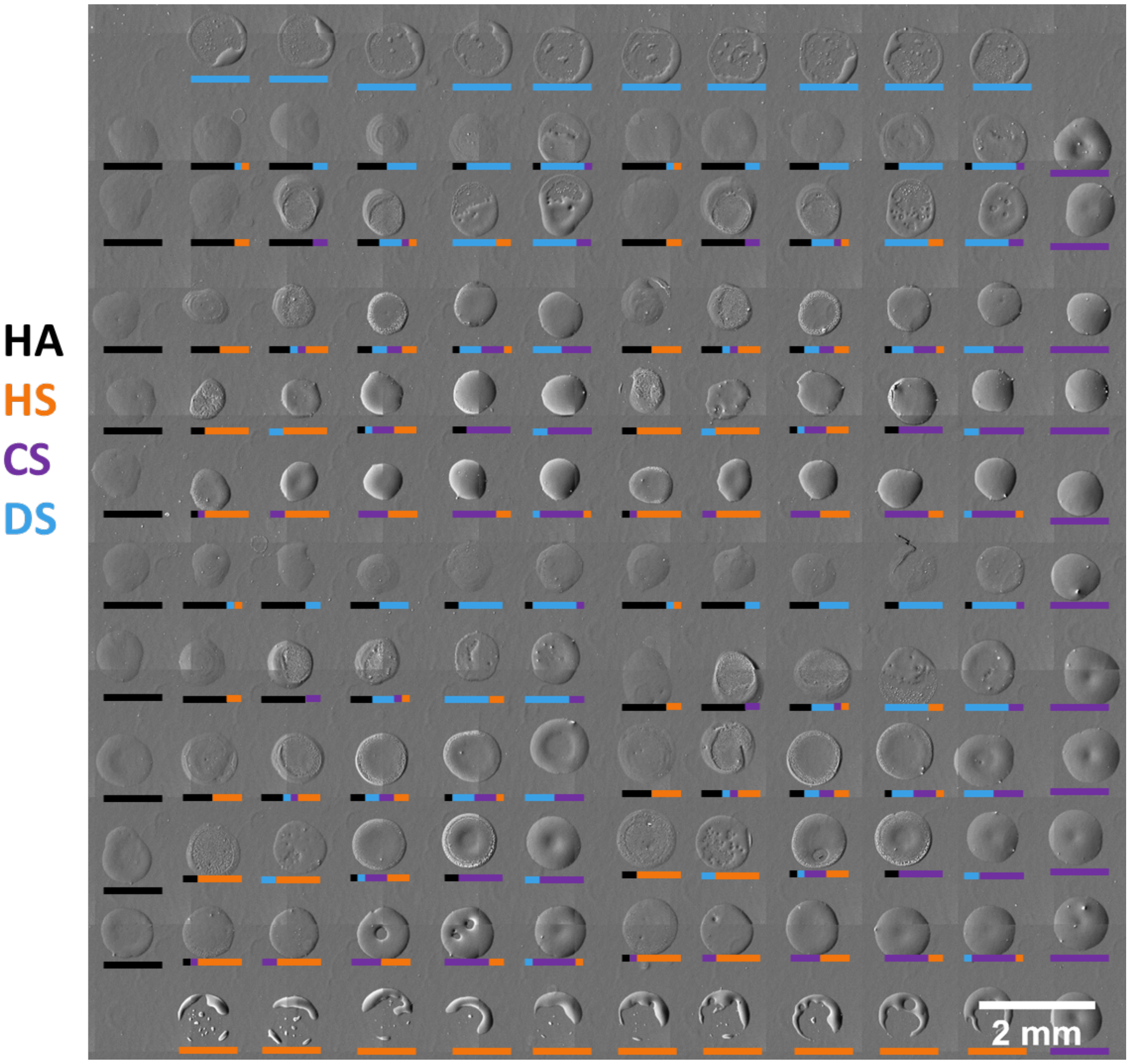

Supplementary Figure 3. Brightfield image of a GAG microarray, pre-printed with15 nL of water and then a total volume of 17 nL of 5 mg/ml GAG solutions, either hyaluronic acid (**HA**), heparan sulphate (**HS**), chondroitin sulphate (**CS**) or dermatan sulphate (**DS**). The bar below each sample indicates the amount of each GAG type added, respective of the fraction coloured. Array printed at 65 % relative humidity. The 5×5 array of mixed GAGs was repeated 4 times.

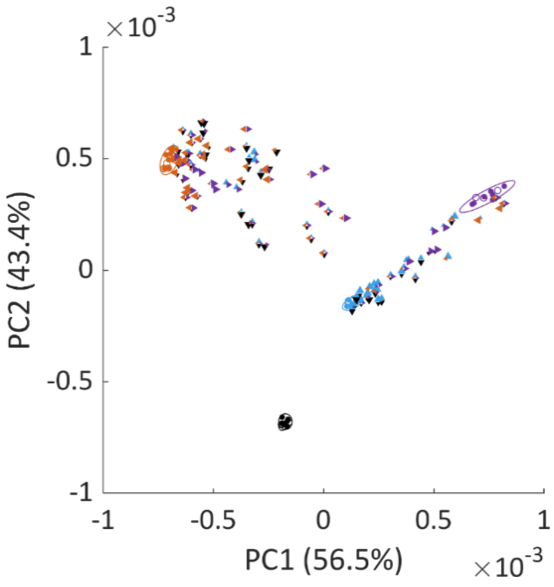

Supplementary Figure 4. Scores plot for PCA analysis of GAG array. Ellipse shows the 95 % confidence limits based upon 7-replicates of each GAG HA (●), HS (●), CS (●), and DS (●). Open circles show test data for 3-replicates of each GAG. Combinations of GAGs are shown as clusters of triangular datapoints, with the size of the triangle indicated the content of each GAG in a sample, respective of the colour. The amount of variance captured by each PC is indicated in the axis title.

Supplementary Table 1. List of key ions associated with PCA of GAG array, and the loading of each ion for PC1 and PC2.

m/z	Possible assignments						PC1	PC2
180.8762	S_4NaNO^+	$CH_2S_4NaO^+$	$C_3HS_4O^+$	$S_3NaNO_3^+$	$H_2S_4NaN_2^+$	$C_2HS_4N_2^+$	0.43	-0.57
61.9676	SNO^-						0.39	0.61
215.9118	$C_5S_3N_2O_2^-$	$C_2H_2S_3NO_5^-$	$CS_2N_2O_7^-$	$C_2H_4S_4N_2O_2^-$	$C_{10}S_3^-$	$C_6H_2S_4N^-$	0.57	-0.38
228.8845	$C_2HS_4N_2O_3^-$	$C_7HS_4O^-$	$C_3HS_4O_4^-$	$C_3HS_3O_6^-$	$C_3H_3S_4NO_3^-$	$C_6HS_4N_2^-$	0.57	0.39

Supplementary Note 3. PCA sparse dataset generation optimisation

In order to optimise the generation of a sparse dataset for PCA a concentration series of heparin samples either derived from porcine mucosa (PM) or bovine lung (BL) were mixed, printed onto an array and analysed by ToF-SIMS. There were 11 technical repeats of each sample, and these samples were split into training and test sets at a training:test ratio of 8:3. Principal component analysis was then done on the full dataset and sparse datasets generated by either recursive feature elimination (RFE) or addition (RFA) using the minimisation of the overlap of 95% confidence ellipses or separation of the distance between the means of datasets for either Euclidean or Mahalanobis geometry as a selection criterion. The total number of features selected was determined as the minimum number of features required to maximise the associated cost function, or to reach an equilibrium state whereupon addition of further features produced no further separation of sample sets. The resulting scores plots are shown in Supplementary Figure 5.

Only the 100 % BL heparin was separated from all other samples using the entire dataset (Supplementary Figure 5a-b), with or without variance scaling, although with variance scaling the separation of 100% BL heparin from all other samples was aligned with PC1 (Supplementary Figure 5a) as opposed to without variance scaling, where the separation was a combination of PC1 and PC2 (Supplementary Figure 5b). PCA of a sparse dataset generated by RFA using the minimisation of overlapping 95% confidence ellipses as a cost function enabled all samples to be separated after consideration of PC1 and PC2 to 95% confidence (Supplementary Figure 5c). However, this PCA model was over-fitted as the test sets did not fall within the 95% confidence ellipses of their respective samples. Random selection of a different training and test set and reapplying the PCA for the same sparse dataset described the samples without over-fitting (all test dataset was within the 95% confidence limit associated with the training set), however, only the 100% and 50% BL heparin samples were successfully separated. This suggested that minimising overlap as a cost function was highly effective at identifying features that could differentiate between samples to 95% confidence but was susceptible to over-fitting due to the selection of features that describe random variance within sample sets rather than features that differentiate between sample sets.

Sparse datasets generated by RFA using the maximisation of the distance between the means of samples sets as a cost function successfully identified features that described variance between the different samples sets, as indicated by reduced overlap of sample sets for the scores plots of PC1 and PC2 (Supplementary Figure 5e-f). This was observed for both Euclidean and Mahalanobis geometry. However, PCA of either dataset was unable to successfully differentiate between the different sample sets to 95% confidence, whereupon no samples sets could be differentiated using PCA of the dataset generated using Mahalanobis geometry (Supplementary Figure 5f), however, the 100% BL heparin sample could be differentiated using the dataset for the Euclidean geometry from all other samples (Supplementary Figure 5e). Although no other sample sets could be differentiated from all other samples, this dataset was able to differentiate between some samples. For example, both the 50% and 0% BL heparin was successfully differentiated from 5 of the other samples concentrations. Moreover, no over-fitting was observed. Therefore, use of the maximisation of the distance between sample set means within Euclidean geometry was able to select features that described the variance between samples, however, separation to 95% confidence was not achieved in all cases.

RFE was also explored as a route to generating a sparse dataset. Use of the minimisation of overlap of 95% confidence limits produced a sparse dataset that did not separate features better than the original dataset. In this case the feature selection was not over fitted. However, this approach selected for features that described different variance within sample sets rather than variance between sample sets, as indicated by the elongation and varied orientation of the ellipses in the scores plot of PC1 and PC2 without achieving separation between the samples (Supplementary Figure 5g).

RFE using the maximisation of the distance between sample sets as a cost function identified a sparse dataset that was not able to differentiate between samples as well as the respective RFA sparse dataset for both Euclidean and Mahalanobis geometries (Supplementary Figure 5h and i).

Of the 140 different ions selected for the 6 different sparse dataset, 28 ions were common to at least 2 different datasets. These ions are listed in Supplementary Figure 5i. A comparison of common ions between sparse datasets is shown in Supplementary Table 1. A maximum of 9 common features were identified when using the distance between sample means in Euclidean geometry as a cost function for RFE and RFA. A similar level of commonality was not observed for sparse datasets generated for the other two costs functions when using RFE or RFA, where only 2 common features were identified for minimising overlap of confidence ellipses and 0 common features were identified for maximising the distance between sample means using Mahalanobis geometry.

As minimising ellipse overlap was the most successful at producing a dataset that could separate samples to 95% confidence using PCA but was prone to overfitting or selection of features that described variance within a dataset, maximising the distance between sample means using Euclidean geometry was initially used as a cost function for RFA to generate a sparse dataset containing only features that describe the separation between sample sets. RFE using the minimisation of ellipse overlap was then applied to generate a secondary sparse model. Using this approach a sparse dataset was produced with optimal separation of samples that was not over-fitted. To further ensure this approach did not overfit data it was applied to generate a sparse dataset for randomly produced data. A total of 1,200 initial features was included, which exceeded the typical numbers of positive and negative ions identified from a ToF-SIMS spectrum. No separation of samples was observed after generating a sparse dataset (Supplementary Figure 6), further confirming the method was not over-fitting the data. This approach was applied for the further generation of sparse datasets.

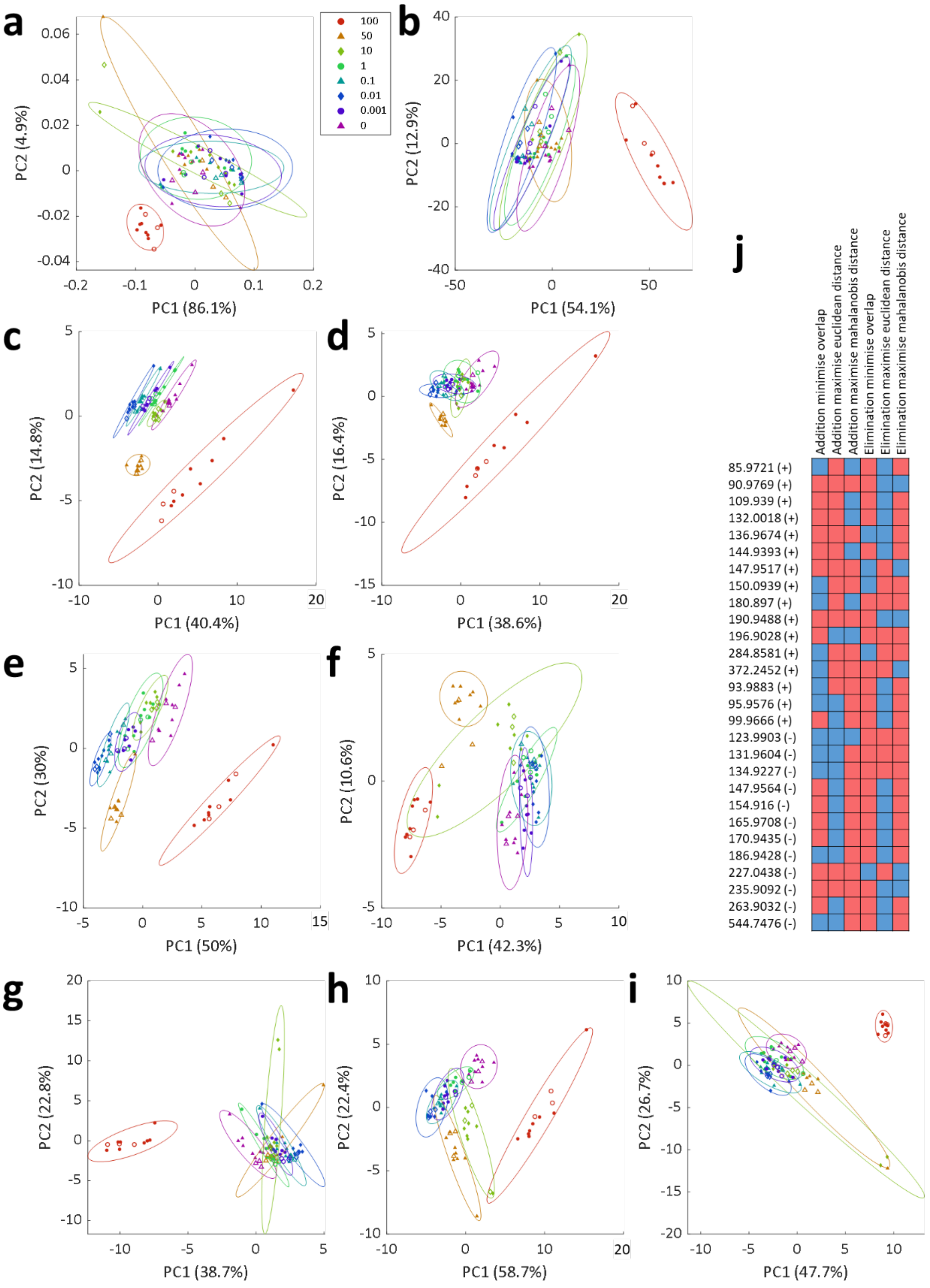

Supplementary Figure 5. Summary of generation of sparse datasets for PCA using various recursive feature selection approaches. PCA scores plot for ToF-SIMS data taken from a concentration series of heparin derived from porcine mucosa spiked with heparin from bovine lung, at weight fractions (%) of 100 (●), 50 (▲), 10 (◆), 1 (●), 0.1 (▲), 0.01 (◆), 0.001 (●), and 0 (▲). Training set closed symbol and test sets open symbols. The 95 % confidence ellipse is drawn around the samples sets, as calculated from the training set data only. The variance captured by each principal component is shown on the axis title. Scores are shown for (**a**) all variables (legend shown applies to **a-i**), (**b**) variance scaled, recursive feature addition (**c-f**) with a selection criteria of (**c**) minimising ellipse overlap, (**d**) same dataset as **c** with a different training and test set, (**e**) maximising Euclidean distance between the mean of each sample set, (**f**) maximising the Mahalanobis distance between the mean of each sample set; and recursive feature elimination with a selection criteria of (**g**) minimising ellipse overlap, (**h**) maximising Euclidean distance between the mean of each sample set, (**i**) maximising the Mahalanobis distance between the mean of each sample set. (**j**) List of the m/z values for ions selected for multiple sparse datasets. The presence of an ion in a particular dataset is indicated as present (red) and absent (blue). A further comparison of the ions selected for the different sparse datasets is shown in Supplementary Table 2.

Supplementary Table 2. Comparison of ions common to different sparse datasets generated using different selection criteria.

	Addition minimise overlap	Addition maximise Euclidean distance	Addition maximise Mahalanobis distance	Elimination minimise overlap	Elimination maximise Euclidean distance	Elimination maximise Mahalanobis distance
Addition minimise overlap	28	6	3	2	5	1
Addition maximise Euclidean distance		20	2	0	9	0
Addition maximise Mahalanobis distance			30	0	4	0
Elimination minimise overlap				35	1	2
Elimination maximise Euclidean distance					30	3
Elimination maximise Mahalanobis distance						40

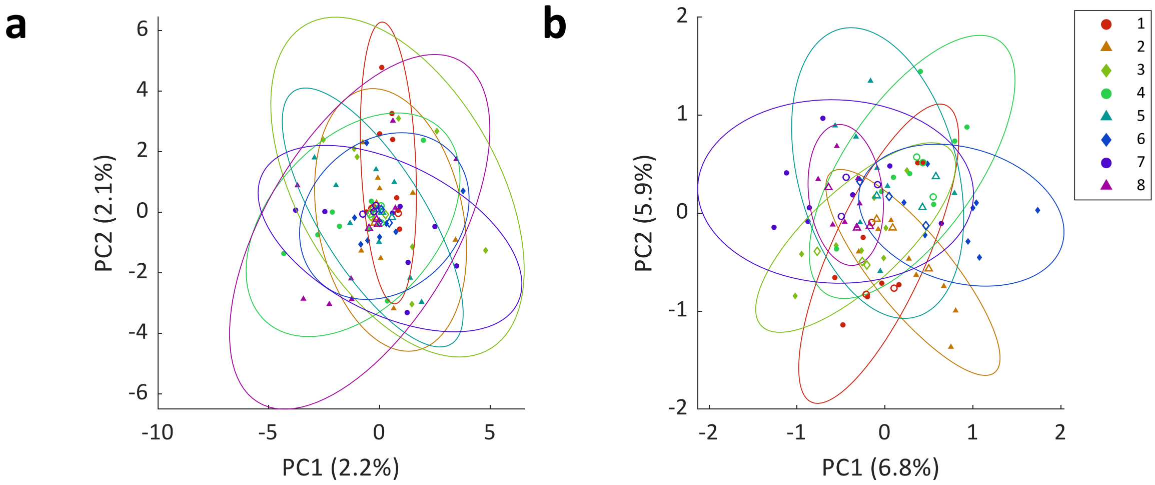

Supplementary Figure 6. PCA of a randomly generated dataset. Training dataset (technical replicates) shown as closed circle and test set shown as open circle. Ellipses are 95 % confidence limits. Variance captured by each PC shown in the axis heading. (**a**) Non-sparse dataset, variance scaled, (**b**) PCA with sparse dataset.

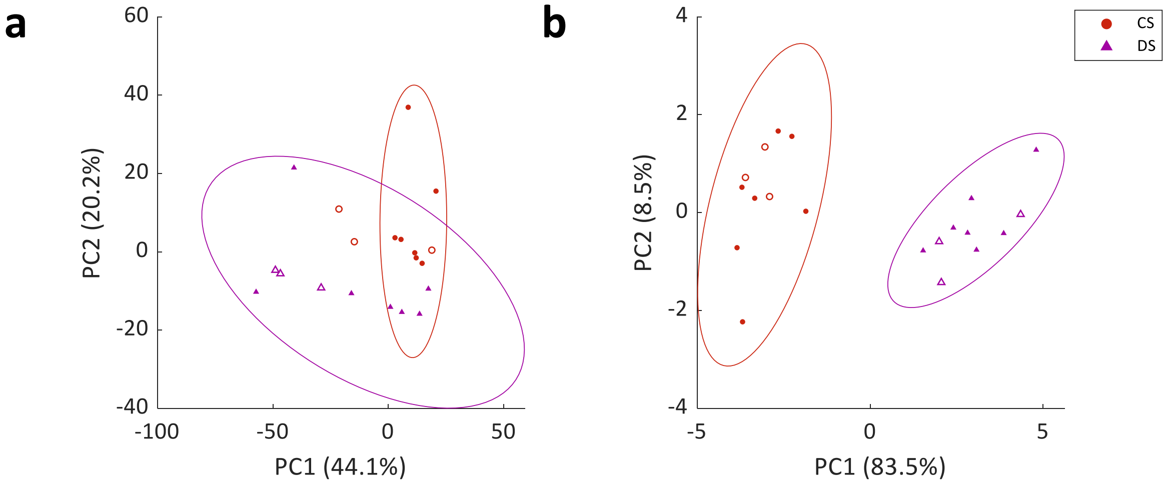

Supplementary Figure 7. PCA of biological replicates of CS (red circles) and DS (purple triangles). Training dataset (technical replicates) shown as closed circle and test set shown as open circle. Ellipses are 95 % confidence limits. Variance captured by each PC shown in the axis heading. (**a**) Non-sparse dataset, variance scaled, (**b**) PCA with sparse dataset.

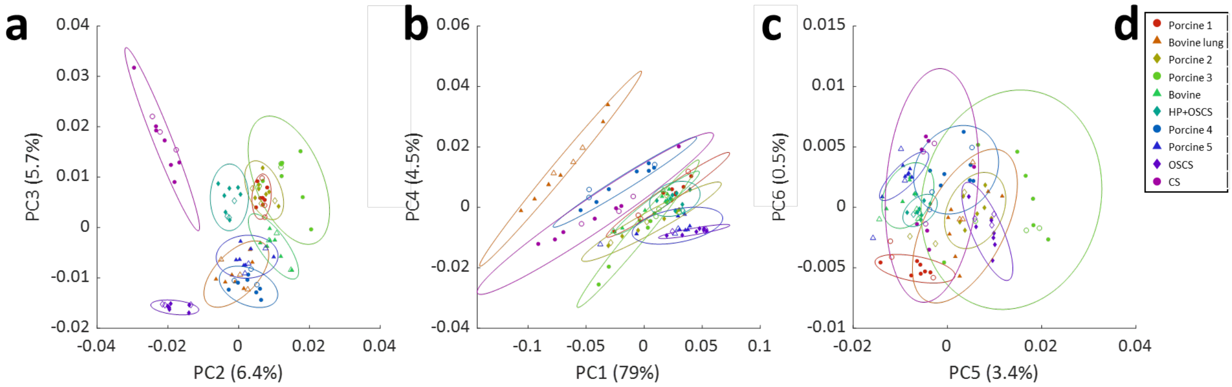

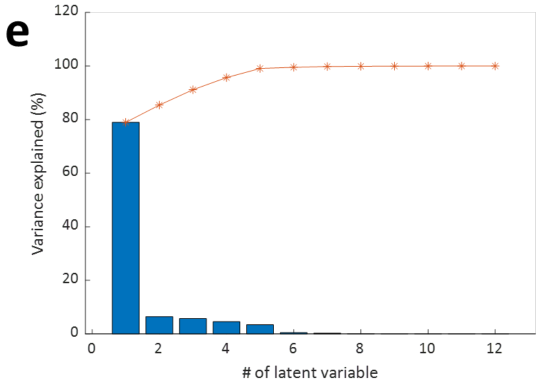

f

#	m/z	Possible assignments		
1	58.0712	$C_3H_8N^+$		
2	62.9811	$HSNO^+$		
3	70.9881	C_2HSN^+		
4	71.9834	$C_2O_3^+$	CSN_2^+	
5	78.9558	HS_2N^+	PO_3^+	
6	90.9769	$CHSNO_2^+$	$C_2H_3SO_2^+$	$C_2H_3S_2^+$
7	148.9341	$C_3HS_2O_3^+$	$C_2HS_2N_2O_2^+$	
8	164.9182	$C_3HS_2O_4^+$		
9	180.897	$C_3HS_2O_5^+$		
10	63.9627	SO_2^-	SO_2^-	
11	79.9648	$H_2S_2N^-$	C_4S^-	SO_3^-
12	96.9646	HSO_4^-		

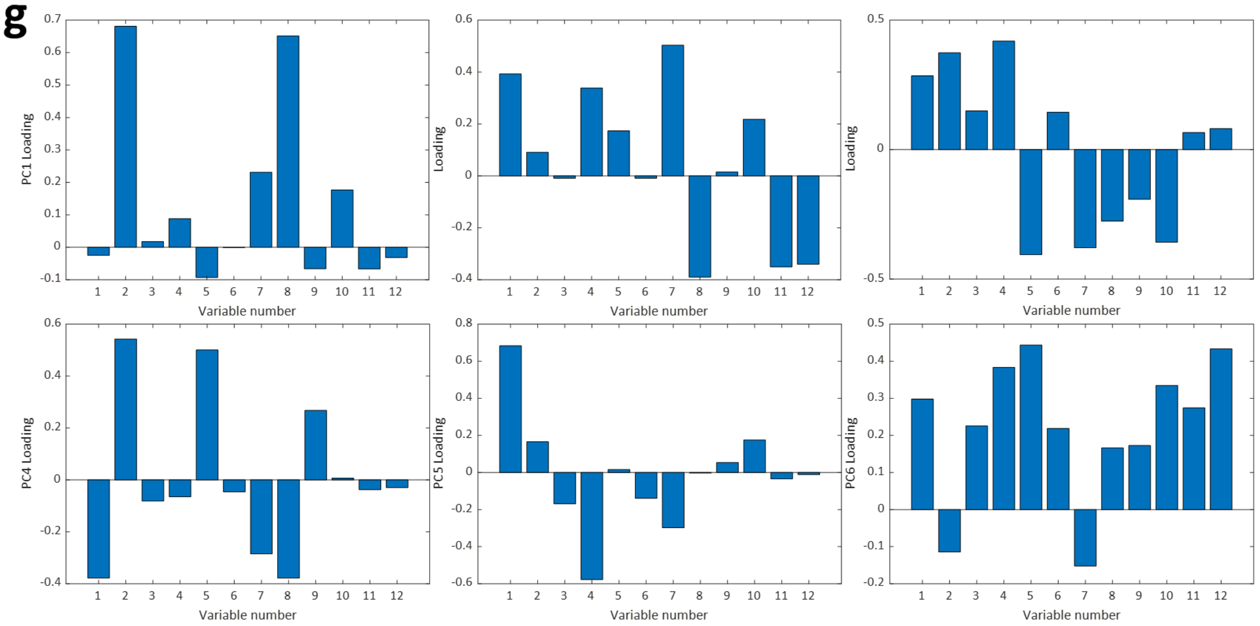

Supplementary Figure 8. Summary of PCA of different heparin samples and CS. (**a-c**) Score plots for PCs 1-6. Training set (n=7) shown as solid markers, whilst test set (n=3) shown as non-filled marker. 95% confidence ellipses shown for each sample type. (**a**) PC2 vs PC3. This combination was shown as it showed the best separation between different CS and heparin samples. PC2 predominately captured variance between heparin and CS samples. PC3 predominately captured variance between different heparin samples. (**b**) PC1 vs PC4, where PC1 predominately captured variance within replicates and PC4 predominately captured variance between the bovine lung derived heparin with the other heparin samples. (**c**) PC5 vs PC6. (**d**) legend of the different samples shown in **a-c**. (**e**) The cumulative (*) and individual (■) variance capture for each latent variable. (**f**) The ions identified for PCA using recursive feature elimination with possible chemical assignments (<100 ppm deviation). (**g**) Loadings for each PC for x-variables listed in **f**. Loadings plots are organised for PCs 1-6 left to right, top down.

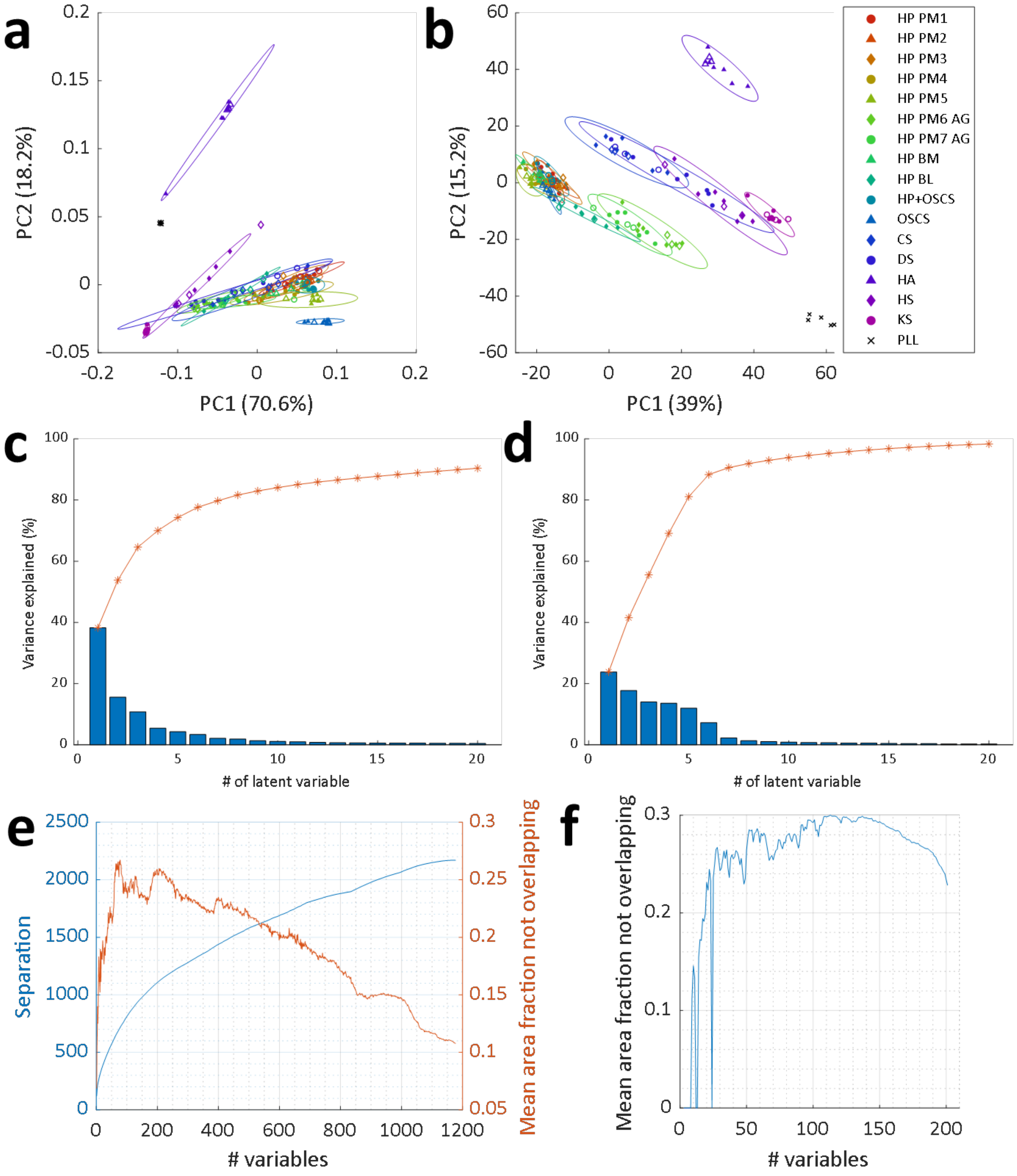

Supplementary Figure 9. Optimisation of the sparse dataset for 16 GAG samples. (**a-b**) Scores plots for PC1 and PC2 using (**a**) data mean centred only, and (**b**) mean-centred and variance scaled. (**c-d**) Scree plot showing the variance captured for each PC (bars) and the cumulative variance (line), for PCA with (**c**) all variables, (**d**) sparse dataset. Selection of number of latent variables used was determined by fitting a linear curve (shown as a dashed line) to the variance explained curve for high numbers of latent variables (15-20) and selecting where the variance explained departed from linearity with reduced numbers of latent variables. (**e**) Separation of the means of each sample set across 6 PCs for varied number of variables using RFA. (**f**) Mean area fraction non-overlapping for confidence ellipses considering PCs 1-6, for PCA conducted with recursive feature elimination. Plot reduced to 0 when further reduction in variable number caused training set samples to fall outside the respective confidence ellipse generated from the scores plots of the training set.

Supplementary Table 3. List of the 48 ions comprising the sparse dataset used for PCA of ToF-SIMS spectra of GAGs (Figure 2). Possible assignments ordered from smallest to largest deviation are shown (<75 ppm), and the loadings associated with PCs 1-6 are indicated according to the intensity scale shown on the right.

m/z	Possible Assignments			PC Loadings					
	1	2	3	1	2	3	4	5	6
29.9963	NO^-								
45.9778	SN^+								
46.07	$C_2H_8N^+$								
55.0204	$C_3H_3O^+$								
59.0474	$C_3H_7O^+$								
74.0619	$C_3H_8NO^+$								
77.9691	SNO_2^-	$CH_2SO_2^-$	$CH_2S_2^-$						
85.9699	C_2SNO^-	$C_3H_2SO^-$							
90.9769	$CHSNO_2^+$	$C_2H_3SO_2^+$	$C_2H_3S_2^+$						
99.9666	$C_3SO_2^-$	$C_3O_4^-$							
100.9682	$C_3HSO_2^-$								
121.994	$C_2H_4SNO_3^-$	$C_6H_2O_3^-$	$C_3H_6SO_3^-$						
129.9536	$C_4H_2S_2O^+$	$C_3SNO_3^+$	$C_4H_2SO_3^+$						
135.0967	$C_6H_{15}O_3^+$	$C_5H_{13}NO_3^+$	$C_6H_{15}SO^+$						
137.9934	$C_6H_2O_4^-$	$C_3H_6SO_4^-$	$C_2H_4SNO_4^-$						
139.971	$C_2H_4SO_5^-$	$C_5H_2SNO_2^-$							
149.9826	$C_4H_6S_2O_2^-$	$C_3H_4SNO_4^-$	$C_7H_2SO_2^-$						
159.9653	$C_5H_4S_2O_2^-$	$C_4H_2SNO_4^-$	$C_4H_2S_2NO_2^-$						
164.9818	$C_4H_5SO_5^-$	$C_7H_3SNO_2^-$	$C_3H_3SNO_5^-$						
176.9334	$C_4HS_2O_4^-$	$C_4HSO_6^-$	$C_4H_3S_2NO_3^-$						
177.9276	$C_4H_2S_2O_4^-$	$C_7S_2NO^-$	$C_3SNO_6^-$						
206.9042	$C_4HS_2NO_5^+$								
211.0227	$C_9H_7O_6^-$	$C_{10}H_{11}S_2O^-$	$C_9H_9SNO_3^-$						
213.9176	$C_6S_2NO_4^+$	$C_7H_2S_2O_4^+$	$C_6SNO_6^+$						
217.9149	$C_5S_2NO_5^-$	$C_6H_2S_2O_5^-$							
226.1225	$C_{12}H_{18}O_4^+$	$C_{12}H_{20}SNO^+$	$C_{11}H_{16}NO_4^+$						
227.0438	$C_{10}H_{11}SO_4^-$	$C_{13}H_7O_4^-$	$C_{10}H_{11}O_6^-$						
255.8892	$C_4S_2O_9^-$	$C_8S_2O_6^-$	$C_4H_2S_2NO_8^-$						
262.8837	$C_7H_3S_4O_3^-$	$C_{10}HS_4N^-$	$C_6HS_3NO_5^-$						
276.8951	$C_7HS_2O_8^-$	$C_6HS_2N_2O_7^-$	$C_4H_5S_2O_{10}^-$						
294.8605	$C_{10}HS_4NO_2^-$	$C_6HS_3NO_7^-$	$C_{11}H_3S_4O_2^-$						
306.8474	$C_7HS_4NO_5^+$	$C_8H_3S_4O_5^+$	$C_{11}HS_4NO_2^+$						
307.8528	$C_7H_2S_4NO_5^+$	$C_{10}S_4N_2O_2^+$	$C_8H_4S_4O_5^+$						
308.8627	$C_7H_3S_4NO_5^+$	$C_{10}HS_4N_2O_2^+$	$C_8H_5S_4O_5^+$						
316.8485	$C_9H_3S_6N^-$	$C_9HS_6O^-$	$C_8HS_5N_2O_2^-$						
317.8804	$C_9H_2S_4O_5^-$	$C_9H_4S_5NO_2^-$	$C_8H_2S_4N_2O_4^-$						
318.8628	$C_9H_3S_5O_3^-$	$C_8HS_5NO_3^-$	$C_8HS_4NO_5^-$						
324.8391	$C_7HS_4O_7^+$								
361.2073	$C_{21}H_{31}SNO_2^+$	$C_{18}H_{33}SO_5^+$	$C_{17}H_{31}NO_7^+$						
374.1602	$C_{20}H_{24}NO_6^-$	$C_{21}H_{28}S_2NO^-$	$C_{17}H_{26}O_9^-$						
374.7765	$C_{10}HS_6NO_3^-$	$C_{11}H_3S_6O_3^-$	$C_7H_3S_5O_8^-$						
375.2504	$C_{18}H_{35}N_2O_6^+$	$C_{23}H_{35}O_4^+$	$C_{19}H_{37}SNO_4^+$						
385.8292	$C_7H_2S_6N_2O_5^-$	$C_{12}H_2S_6O_3^-$	$C_8H_4S_6NO_5^-$						
398.2472	$C_{22}H_{38}SO_4^+$	$C_{17}H_{38}SN_2O_6^+$	$C_{18}H_{38}O_9^+$						
399.2801	$C_{22}H_{41}SNO_3^+$	$C_{25}H_{37}NO_3^+$	$C_{22}H_{39}O_6^+$						
401.8241	$C_7H_2S_6N_2O_6^-$	$C_8H_6S_8N_2O^-$	$C_8H_2S_5O_9^-$						
538.2848	$C_{28}H_{44}SNO_7^-$	$C_{24}H_{44}NO_{12}^-$	$C_{25}H_{48}S_2NO_7^-$						
538.7121	$C_{13}HS_7NO_9^-$	$C_{14}H_3S_7O_9^-$	$C_{13}HS_6NO_{11}^-$						

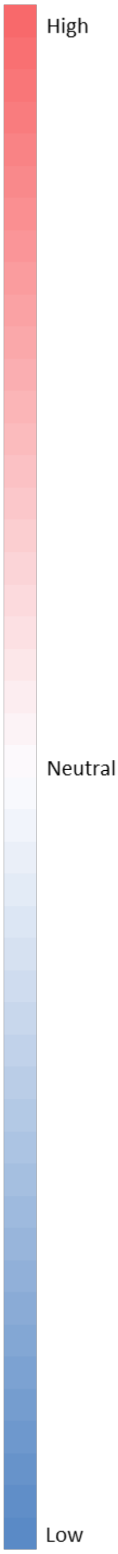

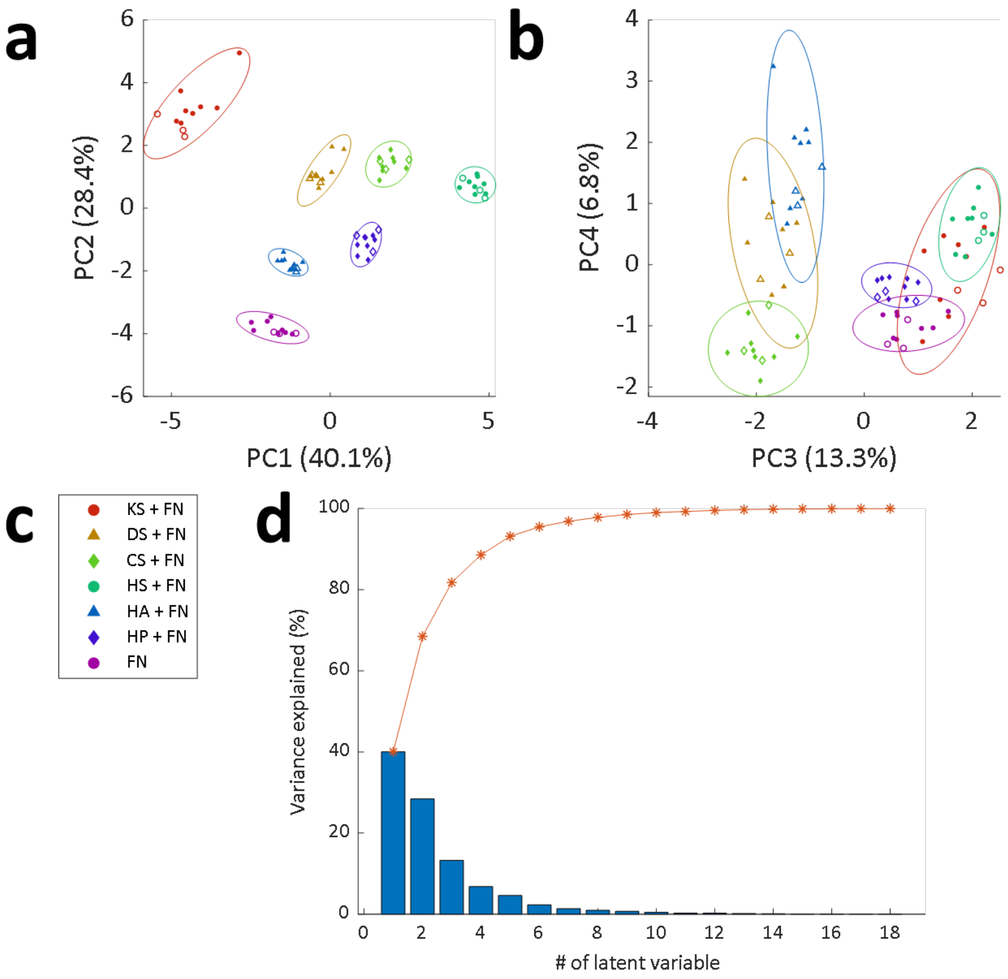

Supplementary Figure 10. Summary of PCA of different GAG samples in fibronectin. (**a-b**) Score plots for PCs 1-4. Training set (n=8) shown as solid markers, whilst test set (n=3) shown as non-filled marker. 95% confidence ellipses shown for each sample type. (**c**) legend of the different samples shown in **a-b**. (**d**) The cumulative (*) and individual (■) variance capture for each latent variable.

Supplementary Table 4. List of the 18 ions comprising the sparse dataset used for PCA of ToF-SIMS spectra of GAGs in fibronectin (Supplementary Figure 10). Possible assignments ordered from smallest to largest deviation are shown (<75 ppm), and the loadings associated with PCs 1-2 are indicated according to the intensity scale shown on the right.

m/z	Possible Assignments			PC Loadings	
	1	2	3	1	2
19.0190	H_3O^+				
30.0342	CH_4N^+				
58.0050	$C_2H_2O_2^-$				
60.9742	$CHSO^-$				
71.9834	$C_5H_{11}^+$				
87.9758	C_2HSNO^+	$C_3H_3SO^+$			
90.9954	$CH_3SN_2O^-$	$CHNO_4^-$	$C_2H_3O_4^-$		
95.0192	$C_5H_5NO^-$	$C_5H_3O_2^-$			
99.9666	$C_3SO_2^-$	$C_3HPO_2^-$	$C_3H_2SNO^-$		
100.9682	$C_3HSO_2^-$				
120.9449	$C_2HS_2O_2^-$				
122.0743	$C_8H_{11}N^+$	$C_9H_{13}^+$	$C_5H_{13}O_3^+$		
132.9841	C_7HSO^-	$C_3H_3SNO_3^-$			
142.9466	$C_8H_{14}O_2^+$				
165.1427	$C_7HS_2O^+$	$C_3HSO_6^+$	$C_3HS_2O_4^+$		
167.9270	$C_6SO_4^-$	$C_6H_2S_2NO^-$	$C_2H_2SNO_6^-$		
174.9900	$C_5H_5S_2NO_2^-$	$C_5H_3SO_5^-$	$C_9H_3SO_2^-$		
256.2452	$C_{13}H_{26}N_3O_2^-$	$C_{10}H_{24}N_8^-$	$C_{14}H_{28}N_2O_2^-$		

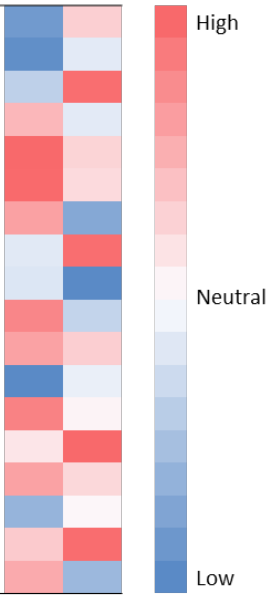

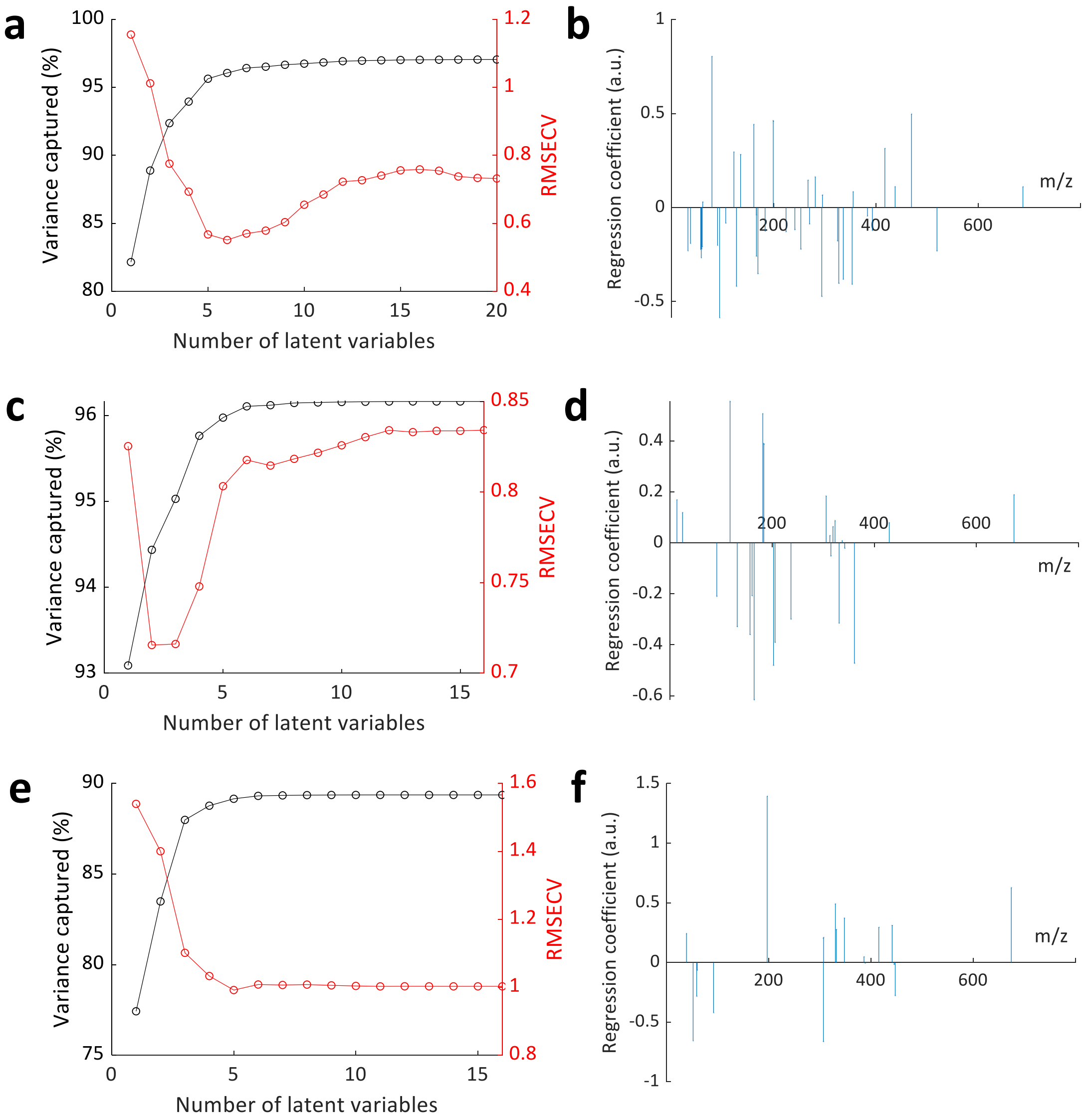

Supplementary Figure 11. PLS regression analysis. (**a, c, e**) Comparison of the variance captured and the root mean square error of cross validation for varied number of latent variables (LV), and (**b, d, f**) the regression coefficients determined for the PLS model for the features selected by LASSO. Samples analysed were heparin PM spiked with (**a**-**b**) OSCS (LV = 6, features = 40), (**c**-**d**) heparin BM (LV = 2, features = 24) and (**e**-**f**) heparin BL (LV = 5, features = 18).

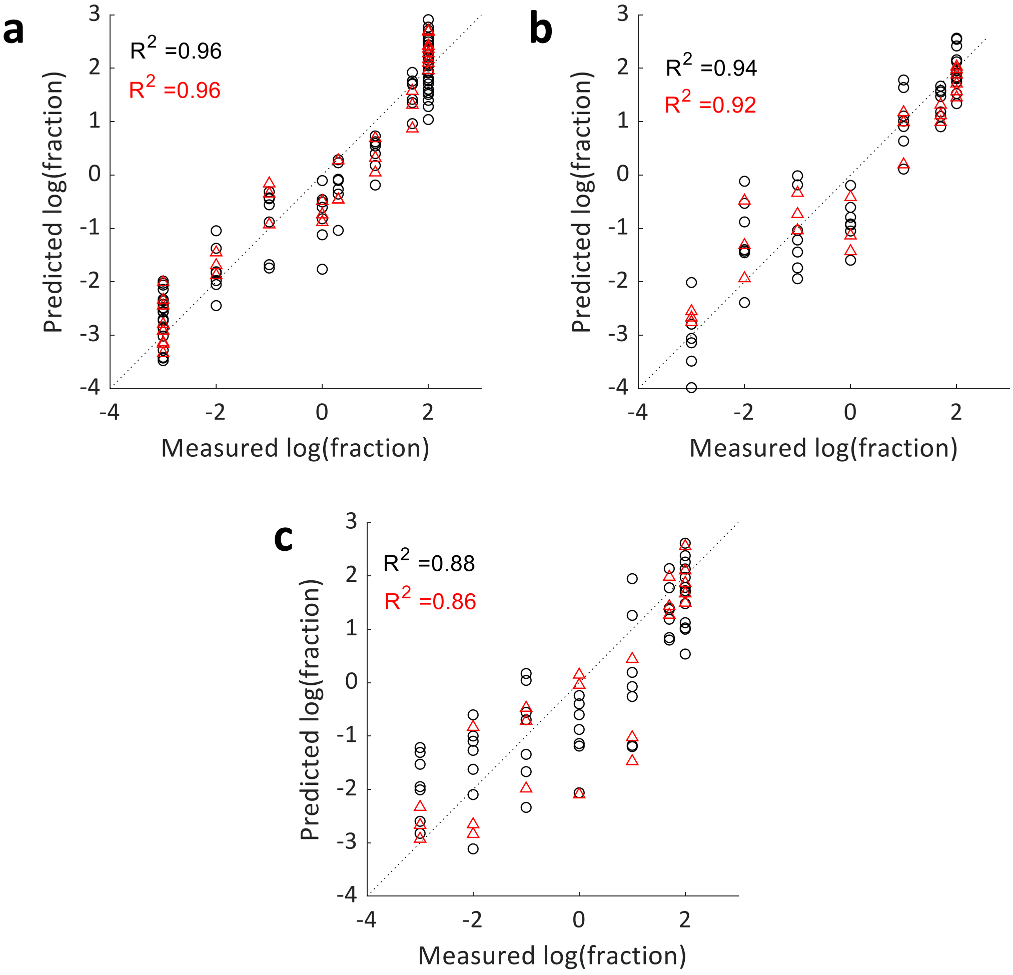

Supplementary Figure 12. PLS measured versus predicted values for heparin (HP) PM spiked with (**a**) OSCS, (**b**) HP BM and (**c**) HP BL, showing training (○) and test sets (△). The y=x line is drawn as a guide. Coefficient of determination values (R^2) for the y=x line are shown for the training (black) and test (red) sets. Measured values were the log of the fraction (%) of contaminant added. Possible assignments and regression coefficients of the ions selected for each model are shown in Supplementary Table 6-8.

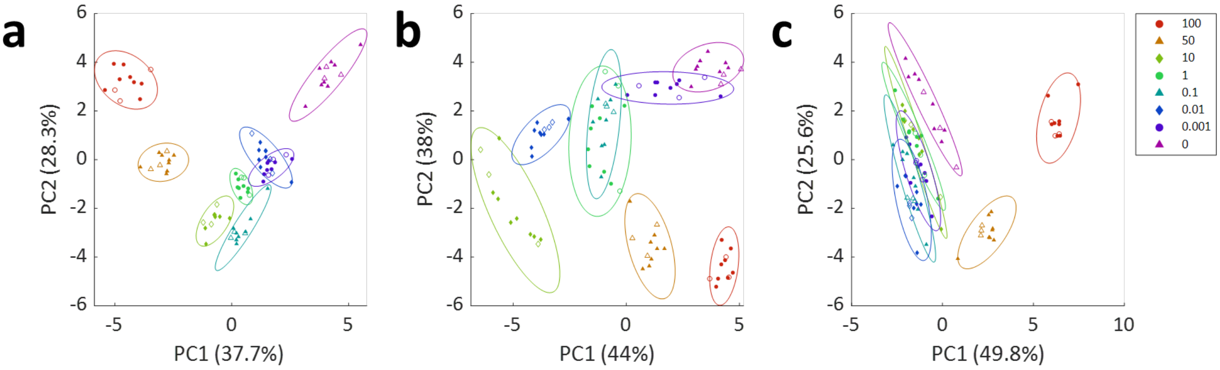

Supplementary Figure 13. PC1 and PC2 scores for heparin PM spiked over a concentration range of 0-100% (wt%) (a) OSCS, (b) heparin BM, or (c) heparin BL, including both training (closed) and test (open) sets. 95% confidence ellipses are shown. Possible assignments and loadings for PC1 and PC2 for the selected features for each dataset are shown in Supplementary Table 5-11. A number of ions likely associated with sulphate groups ($C_3SO_3^-$, $C_6H_{13}S_2O_4^-$) including N-sulphation (SNO_2^-, $CHSNO_2^+$) were present in all sparse datasets. Ions also likely associated with di- and tri-saccharides were also selected ($C_{21}H_{43}O_{10}^+$, $C_{13}H_5S_6O_{12}^-$, $C_8H_5S_2O_8^-$).

Supplementary Table 5. List of the 18 ions comprising the sparse dataset used for PCA of ToF-SIMS spectra of PM heparin spiked with OSCS. Possible assignments ordered from smallest to largest deviation are shown (<75 ppm), and the loadings associated with PCs 1-2 are indicated according to the intensity scale shown on the right.

m/z	Possible Assignments			PC Loadings	
	1	2	3	1	2
39.9948	C_2O^+				
56.9673	C_2HS^+	$C_2HO_2^+$	$C_2H_3NO^+$		
62.9660	$HSNO^-$				
83.9691	C_3SO^-				
100.0338	$C_5H_8S^-$	$C_8H_4^-$	$C_4H_6NO_2^-$		
101.9609	$C_3H_2S_2^-$	$C_2SNO_2^-$			
130.9961	$C_4H_3O_5^-$	$C_7HNO_2^-$	$C_4H_5SNO_2^-$		
131.9608	$C_4H_4S_2O^-$	$C_3H_2SNO_3^-$			
151.9477	$C_2H_2SNO_5^+$	$C_3H_4SO_5^+$			
179.8972	$C_7S_2O_2^-$	$C_6S_2N_2O^-$			
180.8892	$C_7HS_2O_2^+$				
192.9501	$C_4H_3S_2NO_4^+$	$C_4HSO_7^+$	$C_5H_5S_2O_4^+$		
200.2003	$C_{12}H_{26}NO^+$	$C_{13}H_{28}O^+$			
235.9197	$C_6H_4S_3O_4^-$	$C_5H_2S_3NO_4^-$			
266.8394	$C_6H_3S_3O_6^+$	$C_5HS_2NO_8^+$			
293.8860	$C_{11}H_2S_4O_2^-$	$C_7H_2S_3O_7^-$			
348.8865	$C_9H_3S_4NO_6^-$	$C_6H_5S_4O_9^-$	$C_{10}H_5S_4O_6^-$		
455.2817	$C_{25}H_{43}SO_5^+$	$C_{21}H_{43}O_{10}^+$	$C_{24}H_{41}NO_7^+$		

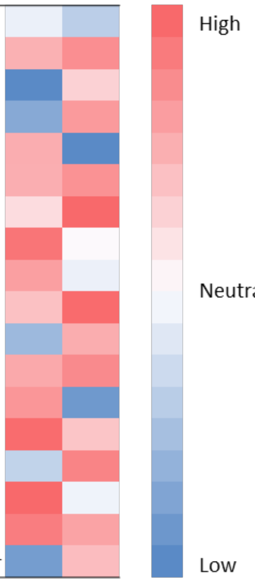

Supplementary Table 6. List of the 15 ions comprising the sparse dataset used for PCA of ToF-SIMS spectra of heparin PM spiked with heparin BL. Possible assignments ordered from smallest to largest deviation are shown (<75 ppm), and the loadings associated with PCs 1-2 are indicated according to the intensity scale shown on the right.

m/z	Possible Assignments			PC Loadings	
	1	2	3	1	2
29.0035	CHO^-				
77.9691	SNO_2^-				
83.9684	C_3SO^-				
90.9769	$CHSNO_2^+$				
96.9564	HSO_4^+				
106.9624	$C_2H_3S_2O^+$	$CHSNO_3^+$			
115.9565	$C_3SO_3^-$				
120.9758	C_6HSO^+	$C_2HO_6^+$	$C_2H_3SNO_3^+$		
137.9302	$CSNO_5^-$				
138.9282	$CHSNO_5^+$				
213.0268	$C_8H_7NO_6^-$	$C_6H_{13}S_2O_4^-$	$C_5H_{11}SNO_6^-$		
222.8808	$C_4HS_2NO_6^-$	$C_5H_3S_2O_6^-$			
230.2627	$C_{14}H_{32}NO^+$	$C_{13}H_{30}N_2O^+$			
324.8391	$C_7H_3S_3NO_8^+$				
544.7476	$C_{13}H_5S_6O_{12}^-$	$C_{16}H_3S_6NO_9^-$	$C_{12}H_5S_6N_2O_{11}^-$		

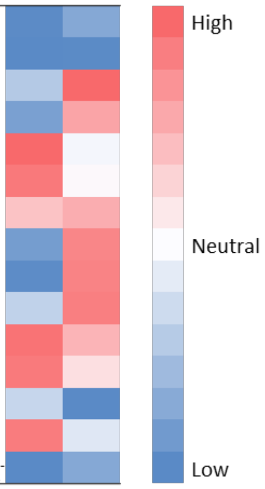

Supplementary Table 7. List of the 25 ions comprising the sparse dataset used for PCA of ToF-SIMS spectra of heparin PM spiked with heparin BM. Possible assignments ordered from smallest to largest deviation are shown (<75 ppm), and the loadings associated with PCs 1-2 are indicated according to the intensity scale shown on the right.

m/z	Possible Assignments			PC Loadings	
	1	2	3	1	2
53.0404	$C_4H_5^-$				
59.0139	$C_2H_3O_2^-$				
77.037	CH_3SNO^+				
93.9883	$CH_2O_5^-$	$CH_4SNO_2^-$			
95.9576	SO_4^-				
100.03	$C_5H_8S^-$	$C_8H_4^-$	$C_4H_6NO_2^-$		
115.9565	$C_3SO_3^-$				
129.9501	$C_3NO_5^-$	$C_4H_2SO_3^-$			
131.9604	$C_3SO_4^-$				
135.9802	$C_3H_6SNO_3^-$	$C_3H_4O_6^-$	$C_7H_4O_3^-$		
138.977	$C_2H_3SO_5^-$	$C_5HSNO_2^-$			
144.0835	$HSNO_6^+$				
147.9564	$C_4H_4SO_4^-$	$C_3H_2NO_6^-$	$C_3H_2SNO_4^-$		
164.9818	$C_8H_5SO_2^-$	$C_4H_5SO_5^-$	$C_4H_5O_7^-$		
186.9428	$C_2H_5SNO_7^-$	$C_6H_3SO_5^-$	$C_6H_3O_7^-$		
223.1637	$C_5H_3S_2O_6^+$	$C_8HS_2NO_3^+$			
246.8976	$C_7H_3SO_8^-$	$C_4H_7S_2O_8^-$	$C_7H_5S_2NO_5^-$		
260.8798	$C_4H_5S_2O_9^-$	$C_7H_3S_2NO_6^-$			
262.8837	$C_7H_3S_2O_7^-$	$C_7H_3SO_9^-$	$C_4H_7S_2O_9^-$		
292.87	$C_7H_3S_2NO_8^-$	$C_8H_5S_2O_8^-$	$C_7H_3SNO_{10}^-$		
318.8628	$C_9H_3S_2O_9^-$				
402.8098	$C_{11}HS_3NO_{10}^-$	$C_{12}H_3S_3O_{10}^-$	$C_{16}H_3S_3O_7^-$		
449.3843	$C_{12}HS_3O_{13}^+$	$C_{16}HS_3O_{10}^+$	$C_9H_5S_3O_{15}^+$		
522.7382	$C_{13}H_3S_3N_2O_{15}^-$	$C_{14}H_5S_3NO_{15}^-$	$C_{17}H_3S_3N_2O_{12}^-$		
686.6418	$C_{32}H_{64}NO_{14}^-$	$C_{31}H_{64}N_3O_{13}^-$	$C_{30}H_{60}N_3O_{14}^-$		

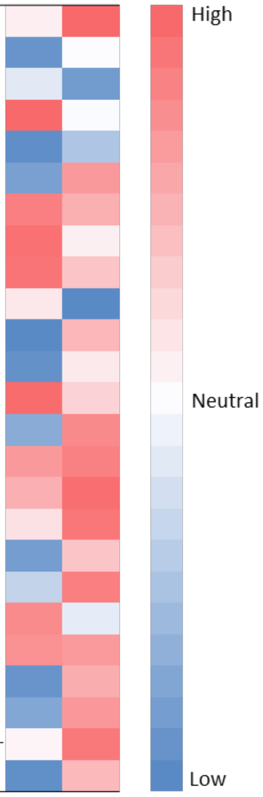

Supplementary Table 8. Anticoagulant profiles of the different heparin samples. Anti-IIa is the antithrombin dependent anti-factor IIa assay, anti-Xa is the antithrombin dependent anti-factor Xa assay and human plasma an activated partial thromboplastin time assay using human plasma. All potencies were assigned relative to the 6th International Standard for Unfractionated Heparin, 07/328. Potency is reported in IU/mg and values in brackets are the standard deviations.

	**Anti-IIa (IU/mg)**	**Anti-Xa (IU/mg)**	**Human Plasma (IU/mg)**
Heparin porcine mucosa 1	209 (13)	204 (9)	194 (8)
Heparin porcine mucosa 2	226 (11)	226 (12)	226 (12)
Heparin porcine mucosa 3	200 (11)	200 (7)	200 (6)
Heparin porcine mucosa 4	183 (9)	184 (13)	185 (8)
Heparin porcine mucosa 5	384 (57)	326 (53)	275 (32)
Heparin bovine mucosa	140 (9)	140 (5)	161 (6)
Heparin bovine lung	154 (8)	141 (7)	129 (8)

Supplementary Table 9. List of the 40 ions comprising the sparse dataset used for PLS regression of ToF-SIMS spectra of porcine mucosa heparin spiked with OSCS (Supplementary Figure 12a). The three possible assignments with the smallest deviation ordered from smallest to largest deviation are shown (<100 ppm), and the regression coefficient for each ion are indicated according to the intensity scale shown on the right. A positive regression coefficient is associated with OSCS whilst a negative regression coefficient is associated with porcine mucosa heparin.

m/z	Possible Assignment 1	2	3	RC
79.02	$C_5H_3O^-$	$NaC_3H_4O^-$		
469.35	$C_{22}H_{49}N_2O_8^+$	$NaC_{23}H_{48}N_3O_5^+$	$C_{21}H_{47}N_3O_8^+$	
198.90	$KC_4S_2O_3^-$	$K_2C_2H_3SNO_3^-$	$NaC_4S_2O_4^-$	
160.94	KC_5SNO^-	$KC_2H_2SO_4^-$	$Na_2C_3HSNO_2^-$	
417.25	$C_{16}H_{37}N_2O_{10}^+$	$KC_{19}H_{40}NO_6^+$	$C_{20}H_{37}SN_2O_5^+$	
121.93	$KNaCSO^-$	$Na_2CSO_2^-$		
135.00	$C_6HNO_3^-$	$C_3H_5SNO_3^-$	$NaC_4H_2NO_3^-$	
281.09	$C_{13}H_{15}NO_6^+$	$C_{10}H_{17}O_9^+$	$NaC_{11}H_{16}NO_6^+$	
266.87	$K_2C_9HS_2O^+$	$K_2C_5HSO_6^+$	$KC_7S_2O_5^+$	
437.25	$C_{22}H_{35}N_3O_6^+$	$C_{20}H_{41}S_2N_2O_4^+$	$C_{19}H_{37}N_2O_9^+$	
687.31	$C_{34}H_{47}SN_4O_9^-$	$NaC_{36}H_{46}N_3O_9^-$	$KC_{33}H_{50}N_3O_{10}^-$	
355.18	$C_{18}H_{29}SNO_4^+$	$C_{14}H_{29}NO_9^+$	$NaC_{16}H_{30}SNO_4^+$	
295.20	$C_{16}H_{27}N_2O_3^+$	$C_{13}H_{31}SN_2O_3^+$	$C_{13}H_{29}NO_6^+$	
61.01	$NaC_3H_2^-$	C_5H^-	$C_2H_5S^-$	
165.92	$Na_2C_2SO_4^+$	$KNaC_2H_2SNO_2^+$	$C_3H_2S_2O_4^+$	
383.26	$C_{20}H_{35}N_2O_5^+$	$C_{17}H_{39}SN_2O_5^+$	$NaC_{18}H_{36}N_2O_5^+$	
105.97	KC_3HNO^-	$C_2H_2SO_3^-$	$CSNO_3^-$	
269.90	$C_8S_3NO_4^-$	$K_2C_5H_4SO_6^-$	$Na_3C_6HS_2O_4^-$	
241.23	$C_{14}H_{27}NO_2^-$			
392.85	$NaC_{11}S_4NO_6^-$	$KC_{11}S_3NO_7^-$	$K_2C_{13}HS_2NO_5^-$	
182.90	$KC_4S_2O_2^+$	$KC_4SO_4^+$	$NaC_4S_2O_3^+$	
223.89	$K_2C_3SNO_4^-$	$K_2C_4H_2SO_4^-$	$KC_6HS_2O_3^-$	
324.84	$K_3C_5H_4S_2O_5^+$	$K_3C_8H_2S_2NO_2^+$	$K_2C_6HS_3NO_4^+$	
37.01	C_3H^-	$NaCH_2^-$		
89.97	KC_3HN^+	$CSNO_2^+$	$C_2H_2SO_2^+$	
60.00	NaC_3H^-	C_5^-	CH_2SN^-	
59.08	$C_3H_9N^+$			
252.90	$NaC_6S_3NO_3^+$	$K_2C_8HSNO_2^+$	$K_2C_5H_5S_2NO_2^+$	
57.03	$C_3H_5O^-$			
519.19	$C_{24}H_{29}N_3O_{10}^-$	$KC_{27}H_{32}N_2O_6^-$	$C_{25}H_{31}SN_2O_8^-$	
32.05	CH_6N^+	$C_4HS_2O_3^-$	$Na_2C_3HS_2N^-$	
165.97	$NaC_5H_3SO_3^-$	KC_8HNO^-	$C_7H_2SO_3^-$	
58.03	$C_2H_4NO^+$			
168.98	$C_{10}HSO^-$	$KC_5H_6SO_2^-$	$NaC_8H_2SO^-$	
335.84	$K_3C_9HS_2NO_2^-$	$K_2C_8H_2S_3O_4^-$	$K_3C_6H_5S_3NO_2^-$	
327.15	$C_{15}H_{23}N_2O_6^+$	$C_{13}H_{29}S_2NO_4^+$	$C_{12}H_{25}NO_9^+$	
353.18	$NaC_{16}H_{28}NO_6^-$	$C_{18}H_{27}NO_6^-$	$C_{14}H_{29}N_2O_8^-$	
126.94	$NaC_2SO_3^-$	$KC_2H_2SNO^-$	$KC_2SO_2^-$	
293.88	$K_2C_{10}H_2S_2NO^-$	$Na_3C_8HS_3O_2^-$	$C_{10}S_4NO_2^-$	
93.99	$NaC_2HNO_2^-$	$C_4NO_2^-$	$CH_4SNO_2^-$	

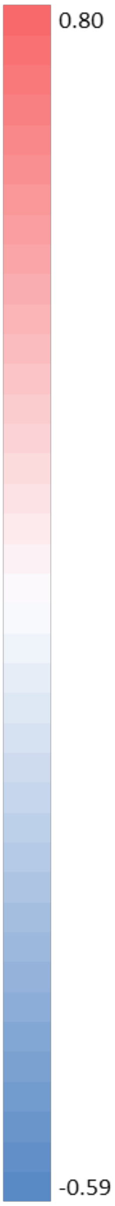

Supplementary Table 10. List of the 24 ions comprising the sparse dataset used for PLS regression of ToF-SIMS spectra of porcine mucosa heparin spiked with bovine mucosa heparin (Supplementary Figure 12b). The three possible assignments with the smallest deviation ordered from smallest to largest deviation are shown (<100 ppm), and the regression coefficient for each ion are indicated according to the intensity scale shown on the right. A positive regression coefficient is associated with bovine mucosa heparin whilst a negative regression coefficient is associated with porcine mucosa heparin.

m/z	Possible Assignment 1	Possible Assignment 2	Possible Assignment 3	RC
117.97	$C_2SNO_3^-$	KC_4HNO^-	$C_3H_2SO_3^-$	
181.94	$C_6S_2NO_2^+$	$NaC_4HS_2NO_2^+$	$C_3H_2S_2O_5^+$	
183.96	$Na_2C_3H_6S_2O_2^+$	$NaC_5H_5S_2O_2^+$	$K_2C_3H_8SNO^+$	
673.37	$C_{31}H_{53}N_4O_{12}^-$	$KC_{34}H_{56}N_3O_8^-$	$C_{32}H_{55}SN_3O_{10}^-$	
305.88	$C_{11}S_4NO_2^+$	$NaC_9HS_4NO_2^+$	$K_2C_8H_4S_2O_4^+$	
14.02	CH_2^-			
25.01	C_2H^-			
323.28	$C_{15}H_{35}N_2O_5^+$			
429.32	$C_{22}H_{43}N_3O_5^+$	$NaC_{21}H_{46}N_2O_5^+$	$C_{22}H_{41}N_2O_6^+$	
319.21	$C_{15}H_{31}SN_2O_3^+$	$NaC_{16}H_{28}N_2O_3^+$	$C_{15}H_{29}NO_6^+$	
313.19	$C_{12}H_{29}N_2O_7^+$	$C_{16}H_{27}NO_5^+$	$NaC_{14}H_{28}NO_5^+$	
337.24	$C_{16}H_{35}NO_6^+$	$C_{15}H_{33}N_2O_6^+$	$C_{19}H_{31}NO_4^+$	
342.24	$NaC_{16}H_{35}N_2O_4^+$	$C_{18}H_{34}N_2O_4^+$	$C_{18}H_{32}NO_5^+$	
315.18	$C_{11}H_{27}N_2O_8^+$	$NaC_{17}H_{26}NO_3^+$	$C_{15}H_{25}NO_6^+$	
160.97	$NaC_3H_6S_2O_2^+$	$NaC_5NO_4^+$	$KC_3H_6SO_3^+$	
92.01	$NaC_3H_3NO^-$	$C_5H_2NO^-$	$KC_4H_5^-$	
237.07	$C_{14}H_9N_2O_2^-$	$C_8H_{15}SNO_5^-$	$C_{11}H_{11}NO_5^-$	
331.23	$C_{17}H_{33}NO_5^+$	$C_{16}H_{31}N_2O_5^+$	$NaC_{14}H_{32}N_2O_5^+$	
131.96	$KC_5HO_2^-$	$C_3H_2S_2NO^-$	$Na_2C_3H_2SO^-$	
156.98	$C_5H_3SNO_3^+$	$NaC_3H_4SNO_3^+$	$C_6H_5S_2O^+$	
205.91	$KNaC_4SO_4^-$	$K_2C_4H_2SNO_2^-$	$KC_3H_3S_2O_4^-$	
361.20	$C_{16}H_{29}N_2O_7^-$	$NaC_{14}H_{30}N_2O_7^-$	$C_{17}H_{31}NO_7^-$	
202.95	$C_6H_5S_3NO^-$	$C_5HSNO_6^-$	$Na_3C_4H_6S_2O^-$	
164.98	$C_7HO_5^-$	$NaC_5H_2O_5^-$	$KC_5H_4NO_3^-$	

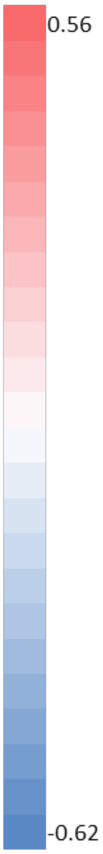

Supplementary Table 11. List of the 18 ions comprising the sparse dataset used for PLS regression of ToF-SIMS spectra of porcine mucosa heparin spiked with bovine lung heparin (Supplementary Figure 12c). The three possible assignments with the smallest deviation ordered from smallest to largest deviation are shown (<100 ppm), and the regression coefficient for each ion are indicated according to the intensity scale shown on the right. A positive regression coefficient is associated with bovine lung heparin whilst a negative regression coefficient is associated with porcine mucosa heparin.

m/z	Possible Assignment			RC
	1	2	3	
196.90	$KC_4S_2NO_2^+$	$K_2C_3H_3SO_3^+$	$KC_5H_2S_2O_2^+$	
674.39	$C_{32}H_{56}N_3O_{12}^-$	$C_{32}H_{56}N_3O_{12}^-$	$C_{31}H_{54}N_4O_{12}^-$	
330.23	$C_{17}H_{32}NO_5^+$	$NaC_{15}H_{33}NO_5^+$	$C_{16}H_{30}N_2O_5^+$	
347.84	$KNa_2C_6HS_3NO_5^-$	$K_3C_7H_5S_3NO_2^-$	$K_2NaC_{10}HS_3NO^-$	
441.35	$C_{24}H_{45}N_2O_5^+$	$NaC_{22}H_{46}N_2O_5^+$	$C_{20}H_{45}N_2O_8^+$	
415.30	$KC_{20}H_{44}N_2O_4^+$	$NaC_{20}H_{42}NO_6^+$	$C_{21}H_{39}N_2O_6^+$	
332.24	$C_{17}H_{34}NO_5^+$	$C_{16}H_{32}N_2O_5^+$	$C_{17}H_{34}SNO_3^+$	
39.02	$NaCH_4^+$	$C_3H_3^+$		
307.23	$C_{15}H_{33}NO_5^+$	$C_{14}H_{31}N_2O_5^+$	$C_{15}H_{33}SNO_3^+$	
386.28	$C_{20}H_{38}N_2O_5^+$	$NaC_{18}H_{39}N_2O_5^+$	$C_{20}H_{36}NO_6^+$	
389.27	$C_{19}H_{37}N_2O_6^+$	$NaC_{17}H_{38}N_2O_6^+$	$C_{19}H_{35}NO_7^+$	
445.34	$C_{23}H_{47}N_3O_5^+$	$C_{23}H_{45}N_2O_6^+$	$NaC_{21}H_{46}N_2O_6^+$	
60.00	NaC_3H^-	C_5^-	CH_2SN^-	
447.33	$C_{22}H_{45}N_3O_6^+$	$C_{23}H_{47}N_2O_6^+$	$C_{22}H_{43}N_2O_7^+$	
58.99	$C_2H_3S^+$	NaC_3^+		
92.01	$NaC_3H_3NO^-$	$C_5H_2NO^-$	$KC_4H_5^-$	
51.99	$NaCHO^+$	C_3O^+		
306.85	$KNaC_7HS_3O_4^+$	$KC_9S_3O_4^+$	$KC_5S_2O_9^+$	

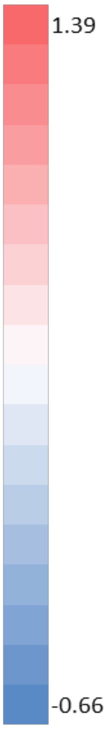

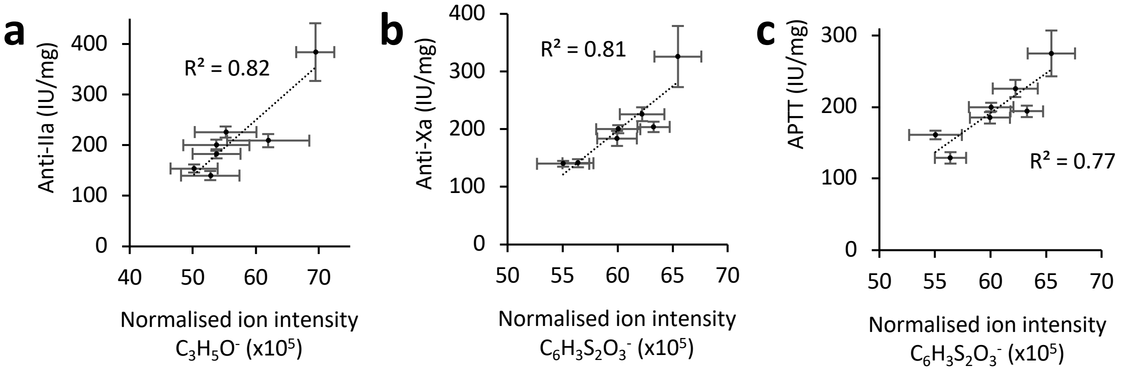

Supplementary Figure 14. Linear correlation between the normalised ion intensity of selected ions with the activity of pharmaceutical grade heparin. Ions were selected that showed significant ($p<0.001$) linear correlations (Pearson's > 0.75) with heparin activity (**a**) Anti-IIa, (**b**) Anti-Xa and (**c**) human plasma.

Supplementary References

1. Wendeln, C.; Heile, A.; Arlinghaus, H. F.; Ravoo, B. J., Carbohydrate Microarrays by Microcontact Printing. *Langmuir* **2010,** *26* (7), 4933-4940.
2. Burzava, A. L. S.; Jasieniak, M.; Cockshell, M. P.; Bonder, C. S.; Harding, F. J.; Griesser, H. J.; Voelcker, N. H., Affinity Binding of EMR2 Expressing Cells by Surface-Grafted Chondroitin Sulfate B. *Biomacromolecules* **2017,** *18* (6), 1697-1704.
3. Charbonneau, C.; Liberelle, B.; Hebert, M. J.; De Crescenzo, G.; Lerouge, S., Stimulation of cell growth and resistance to apoptosis in vascular smooth muscle cells on a chondroitin sulfate/epidermal growth factor coating. *Biomaterials* **2011,** *32* (6), 1591-1600.
4. Chevolot, Y.; Bucher, O.; Leonard, D.; Mathieu, H. J.; Sigrist, H., Synthesis and characterization of a photoactivatable glycoaryldiazirine for surface glycoengineering. *Bioconjugate Chem.* **1999,** *10* (2), 169-175.

5. Erdtmann, M.; Keller, R.; Baumann, H., Photochemical immobilization of heparin, dermatan sulphate, dextran sulphate and endothelial cell surface heparan sulphate onto cellulose membranes for the preparation of athrombogenic and antithrombogenic polymers. *Biomaterials* **1994,** *15* (13), 1043-1048.
6. Kilcoyne, M.; Gerlach, J. Q.; Kane, M.; Joshi, L., Surface chemistry and linker effects on lectin-carbohydrate recognition for glycan microarrays. *Analytical Methods* **2012,** *4* (9), 2721-2728.
7. Robinson, D. E.; Buttle, D. J.; Short, R. D.; McArthur, S. L.; Steele, D. A.; Whittle, J. D., Glycosaminoglycan (GAG) binding surfaces for characterizing GAG-protein interactions. *Biomaterials* **2012,** *33* (4), 1007-1016.
8. Meade, K. A.; White, K. J.; Pickford, C. E.; Holley, R. J.; Marson, A.; Tillotson, D.; van Kuppevelt, T. H.; Whittle, J. D.; Day, A. J.; Merry, C. L. R., Immobilization of Heparan Sulfate on Electrospun Meshes to Support Embryonic Stem Cell Culture and Differentiation. *J. Biol. Chem.* **2013,** *288* (8), 5530-5538.
9. Scurr, D. J.; Horlacher, T.; Oberli, M. A.; Werz, D. B.; Kroeck, L.; Bufali, S.; Seeberger, P. H.; Shard, A. G.; Alexander, M. R., Surface Characterization of Carbohydrate Microarrays. *Langmuir* **2010,** *26* (22), 17143-17155.
10. Yang, J.; Hsieh, P.-H.; Liu, X.; Zhou, W.; Zhang, X.; Zhao, J.; Xu, Y.; Zhang, F.; Linhardt, R. J.; Liu, J., Construction and characterisation of a heparan sulphate heptasaccharide microarray. *Chem. Commun.* **2017,** *53* (10), 1743-1746.
11. Park, T.-J.; Lee, M.-Y.; Dordick, J. S.; Linhardt, R. J., Signal amplification of target protein on heparin glycan microarray. *Anal. Biochem.* **2008,** *383* (1), 116-121.
12. Rogers, C. J.; Clark, P. M.; Tully, S. E.; Abrol, R.; Garcia, K. C.; Goddard, W. A., III; Hsieh-Wilson, L. C., Elucidating glycosaminoglycan-protein-protein interactions using carbohydrate microarray and computational approaches. *Proc. Natl. Acad. Sci. USA* **2011,** *108* (24), 9747-9752.
13. Shipp, E. L.; Hsieh-Wilson, L. C., Profiling the sulfation specificities of glycosaminoglycan interactions with growth factors and chemotactic proteins using microarrays. *Chem. Biol.* **2007,** *14* (2), 195-208.
14. Stowell, S. R.; Arthur, C. M.; McBride, R.; Berger, O.; Razi, N.; Heimburg-Molinaro, J.; Rodrigues, L. C.; Gourdine, J.-P.; Noll, A. J.; von Gunten, S.; Smith, D. F.; Knirel, Y. A.; Paulson, J. C.; Cummings, R. D., Microbial glycan microarrays define key features of host-microbial interactions. *Nat. Chem. Biol.* **2014,** *10* (6), 470-476.
15. Zhang, R.; Liberski, A.; Khan, F.; Diaz-Mochon, J. J.; Bradley, M., Inkjet fabrication of hydrogel microarrays using in situ nanolitre-scale polymerisation. *Chem. Commun.* **2008,** (11), 1317-1319.
16. Vanden Eynde, X.; Bertrand, P.; Penelle, J., "Matrix" effects in ToF-SIMS analyses of styrene-methyl methacrylate random copolymers. *Macromolecules* **2000,** *33* (15), 5624-5633.
